# Integrated geophysical and geochemical methods applied for recognition of acid waste drainage (AWD) from Zn-Pb post-flotation tailing pile (Olkusz, southern Poland)

**DOI:** 10.1007/s11356-020-08195-4

**Published:** 2020-03-04

**Authors:** Jolanta Pierwoła, Marcin Szuszkiewicz, Jerzy Cabala, Krzysztof Jochymczyk, Bogdan Żogała, Tadeusz Magiera

**Affiliations:** 1 Faculty of Natural Sciences, University of Silesia, 60 Będzińska St., PL-41-200 Sosnowiec, Poland; 2 Institute of Environmental Engineering, Polish Academy of Sciences, 34 Skłodowskiej-Curie St., PL-41-819 Zabrze, Poland

**Keywords:** Acid waste drainage (AWD), Zn-Pb flotation tailings, Vadoze zone, Geoelectrical and magnetic methods, SEM, EDS, XRD methods

## Abstract

Long-term underground exploitation of Zn-Pb ores has led to drainage of the area and formation of a huge dumping ground in the form of a pile. In its vicinity, processes of acid drainage have developed as a result of contamination of soils and groundwater. Geochemical transformations of mineral contents of waste can significantly affect physical and chemical properties of the soils and the bedrock. At the prospect of termination of the mining activity in the near future, determining the routes of the pollution migration, ability to monitor acid drainage processes and assessment of the risk of heavy metal pollution are really crucial. The paper presents a proposal for solving this problem by means of geophysical methods: Electrical Resistivity Tomography (ERT), Time Domain-Induced Polarisation (TDIP), Frequency Domain Electromagnetics (FDEM) and shallow-depth magnetometric surveys combined with geochemical investigations. The obtained results of geophysical surveys have been confirmed by geochemical investigations. The applied ERT and TDIP methods make it possible to identify the spread of the zones of pollution around the tailing pile, but their effectiveness depends on humidity of the ground. Soil magnetometry and shallow-depth induction profiling are a good tool to identify the medium contaminated with minerals redeposited by aeolian processes and allow to determine the range of the dust spread from the pile. It has been shown that the range of impact of the geochemical changes around the tailing pile is high and depends not only on directions and dynamics of water flow from the pile but also on aeolian transport.

## Introduction

Large-scale exploitation and processing of Cu and Zn-Pb ores which has lasted for the last 50 years has led to production of waste, which constitutes about 20% of all industrial waste deposited in Poland. In the vicinity of Olkusz near Kraków (southern Poland), about 60 million tons of waste from the flotation of Zn-Pb ore of the Mississippi Valley-Type (MVT) has been deposited in the area of ca. 120 ha.

Geochemical transformations of mineral components of the waste, especially in fine-grained fractions, as well as appreciable share of metal-bearing Fe, Zn and Pb sulphides can have a significant impact on the local ecosystem including bedrock, soil, air, groundwater and its biotic elements. The way and scale of this environmental impact is most often assessed on the basis of mineralogical and geochemical analyses (Alpers et al. 1994; Lefebvre et al. 2001;  Bucby et al. 2003; Cabala et al. 2004; Bauerek et al. 2009, 2013).

The abovementioned transformations significantly influence the physicochemical properties of soils and the bedrock, thus application of geophysical methods, in particular the geoelectric ones, for understanding the paths of pollution migration seems to be quite promising.

Transfer of the sulphide oxidation products: Zn^2+^, Cd^2+^, Pb^2+^, Mg^2+^, Ca^2+^ and SO_4_^2−^ change the electrical conductivity of the rock mass (Schön 2015). Therefore electrical resistivity and electromagnetic methods are successfully used to identify zones of acid drainage related to exploitation and processing of metal ores (Vanhala et al. 2004; Chouteau et al. 2006; Cabala et al. 2008; Iacob and Orza 2008; Gómez-Ortiz et al., 2010; Nearing et al. 2013; Acosta et al. 2014; Olenchenko et al. 2016; Epov et al. 2017) and of hard coal (Ladwig 1983; Pierwola and Kowalska 2012; Pierwoła 2013; Power et al. 2018).

The induced polarisation method is widely used in environmental studies because it enables to distinguish changes in electrical conductivity due to the presence of mineralised water from those caused by the clayey or conductive minerals.

The range and size of redeposition of the dust fraction from waste dumping grounds, not covered with sod, rich in Fe sulphides can be determined by soil magnetometry methods. The weathering processes cause the iron to pass from the paramagnetic forms to the ferri- or antiferrimagnetic forms, which results in an increase in the value of magnetic susceptibility. The research (Rachwał et al. 2017; Magiera et al. 2018) showed a high correlation between the magnetic susceptibility values and Zn, Mn, Cu, As and Sb, the potentially toxic elements (PTEs) characteristic for emissions associated with storage and processing of Zn-Pb ores.

There have not been any comprehensive geophysical and geochemical investigations in the vicinity of the Olkusz pile with post-flotation tailings after Zn-Pb ore processing, which interpretation would allow to identify the range of pollution and to determine the directions of the pollution transport. The aim of the carried out research has been to assess possibility of application of the set of research methods to identify zones of pollution spreading into the soil and rock mass in the vicinity of the tailing pile. Determination of the extend and dynamics of sulphate ions and heavy metal (HM) transfer is particularly important in case of termination of the exploitation, discontinuation of water mine drainage (WMD) and gradual reconstruction of the original hydrological system, which poses a real threat of pollution to the Triassic part of the karst-fissure drinking water reservoir.

## Materials and methods

### Area of study

**Fig. 1:**
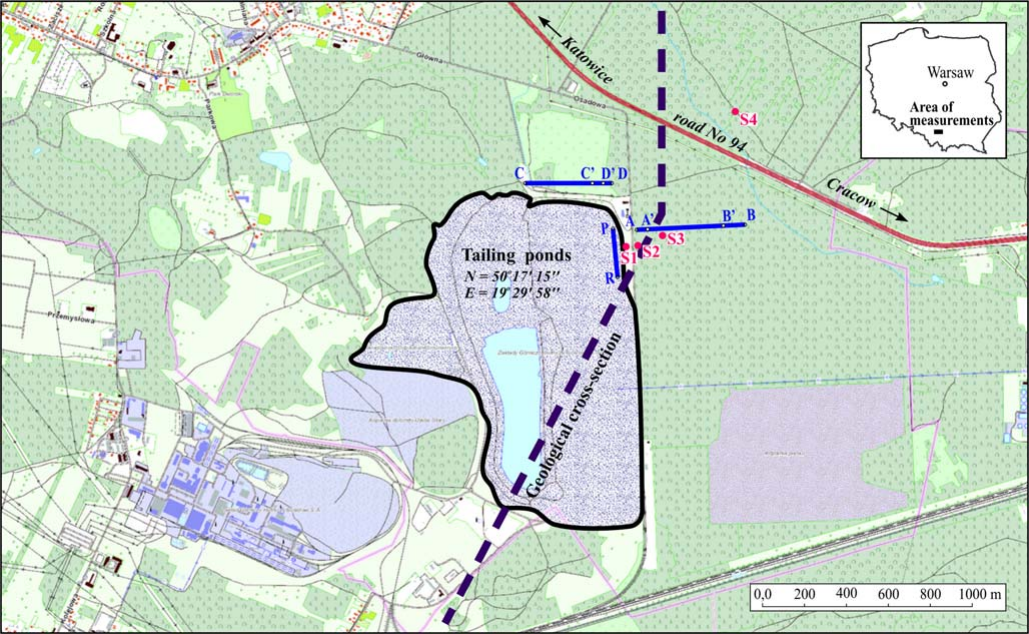
Location of the investigated area with the lines of geophysical measurements (blue lines) and sampling areas. S1 At a distance of 0 m from tailing pile. S2 50–200 m from tailing pile. S3 300–400 m from tailing pile. S4 1000 m from tailing pile (sampling depth 0–0.8 m)

**Fig. 2:**
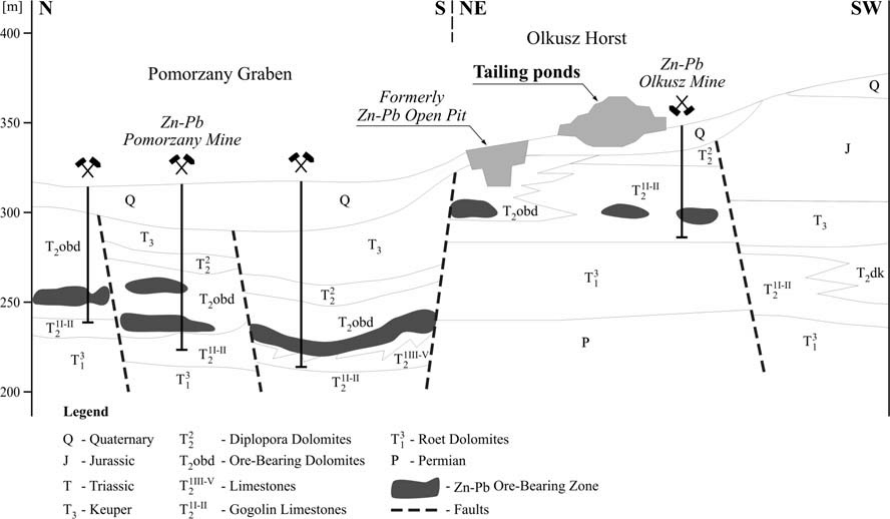
Geological cross section

The Zn-Pb ore tailing dumping ground (Fig. 1) was established in the former sand pit at the end of the 1960s. Protection of the substrate against infiltration of leachate from the dumping ground has been poor due to its location on fluvioglacial sands, fine and medium grained ones with intercalations of gravel, debris, clay, dust and loam (Motyka et al. 2005). The sands were deposited in the Pleistocene in the paleovalley of the Przemsza River, and their thickness varies from several to about 60 m. Under the sandy sediments, there are carbonate Triassic rocks (Fig.2). In the area of the dumping ground, they built the tectonic horst Olkusz-Bolesław (Cabala 2001). The Triassic ceiling is of erosive character. On the edges, there are exposures of various Triassic formations: ore-bearing dolomites, Gogolin limestones and Roethian dolomites. The more complete profile of the Triassic formation isolated by impermeable, silty deposits of the Keuper is represented in trough faults, e.g. the Pomorzany graben (Fig. 2).

A complete soil profile has not developed in the vicinity of the dumping ground. In the area of outcrops of carbonate rocks, soils have the characteristics of initial rendzinas or rendzic leptosols or podzols on fluvioglacial sands (Gruszczyński et al. 1990). In the area of research, there are initial soils (Regosols) and industrial ones (Technosols).

In the hydrogeological system, it is possible to distinguish Quaternary level related to sands, with a filtration coefficient of 2.5 × 10^−4^ m/s, which has been drained by carbonate, karst-fissure formations of the Triassic level. Fissured and cavernous diplopora and ore-bearing dolomites occurring in the upper Triassic are very well permeable (10^−5^–10^−4^ m/s) (Postawa and Motyka 2018). The layers of Olkusz and Gogolin limestones located below contain marl and clay intercalations, hence their permeability is lower—in the order of 10^−7^–10^−5^ m/s.

Over 50 years of the underground exploitation of shallow Zn-Pb ore has resulted in drainage of the Quaternary and Triassic levels. The mines, pumping about 200 m^3^ of water/min, have formed a cone of depression of a diameter of up to 20 km (Motyka et al. 2016; Postawa and Motyka 2018).

### Geophysical investigations

Geophysical investigations were carried out along three profiles in close proximity to the tailing dumping ground (Fig.1). Profile A-B is oriented perpendicularly to the eastern slope of the pile. The C-D profile runs parallel to its northern slope. The third profile P-R, running longitudinally, was delineated on the eastern slope of the pile and served as a reference line.

Along these profiles, the measurements of electrical resistivity (ERT) and induced polarisation (TDIP), with the Lund Imaging System (ABEM); conductometric profilings (FDEM), with conductivity metres EM38-MK2 and EM31-MK2 (Geonics Ltd.); and low-field magnetic susceptibility, using MS2 Bartington susceptibility metre (Bartington Instruments Ltd.) were carried out.

ERT measurements were conducted using the Wenner-Schlumberger array with the smallest distance between the electrodes *a* = 5 m. The array type and geometry were chosen to provide suitable depth of recognition, satisfactory resolution and adequate signal level (Loke, 2019) in dry sands of fluvioglacial origin which was unfavourable for electrical measurements.

Conductometric profilings were made using devices with fixed dipole spacings: 0.5 m, 1.0 m and 3.66 m, which in vertical orientation (VD) enabled recognition to the depths of 0.75 m, 1.5 m and 6 m respectively. The EM surveys have given detailed information from shallow-depth intervals omitted during the ERT. To recognise the anomalous, near-surface intervals of the A-B and C-D lines, identified in EM results, during the induced polarisation measurements, the unit electrode spacing was reduced to *a* = 0.5 m.

For the measurements of volume magnetic susceptibility (κ), which is the ratio of the magnetization to magnetic field (Hunt et al. 1995) , the MS2 Bartington metre with MS2D loop probe was used. This probe (with a diameter of 185 mm) has been designed for outdoor use, in particular, for soil surface measurements with a maximum penetration depth of 10 cm; however, 90% of the signal came from the layer not deeper than 6 cm (Dearing 1999; Lecoanet et al. 1999). At each individual location point, geographic coordinates were determined using the GPS system, and 11 MS2D individual readings of soil magnetic susceptibility, evenly distributed within a circle of about 2-m radius, were made. The final κ value was the average of 11 data after statistical evaluation and elimination of outliers.

Collected soil samples (S1–4) for a laboratory magnetic measurements were air dried, homogenised, weighed and placed in plastic container of 10 cm^3^. Measurements of κ were performed using a MS2 Bartington metre with a MS2B sensor at low (465 Hz) and high (4650 Hz) frequencies. Using the κ value obtained and the sample density, the mass magnetic susceptibility (χ) value was calculated. Additionally, a percentage frequency-dependent magnetic susceptibility (χ_FD%_) was computed (χ_FD%_ = (χ_465Hz_–χ_4650Hz_)/ χ_465Hz_) (Dearing, 1999).

### Mineralogical analyses

Electron-probe microanalyses and observations were performed using an Environmental Scanning Electron Microscope (Philips XL 30) with an EDS (energy dispersive X-ray spectrometer) analyser. The investigated material (waste, soil and sand) was fixed to carbon tapes (1 × 1.5 cm) placed on aluminium stubs. For each sample, a few hundred representative particles were placed onto the carbon adhesive base and examined using a stereo microscope. Thirty samples of wastes, topsoil and more deeply sandy sediments were viewed uncoated using a SEM with a back-scattered electron (BSE) detector (Centaurus). Eighty qualitative microanalyses (EDS) of minerals of all the 30 samples were carried out using an attached EDS (Sapphire) (15-kV accelerating voltage, environmental mode and 40-Pa H_2_O pressure). Ninety BSE images of soil and waste material of all the 30 samples were registered to document the analysed material. The presence of characteristic peaks of some elements in the EDS spectra enabled determination of the elemental compositions.

X-ray diffraction analyses (XRD) of the mineral components were made on soil samples collected on the waste pile (S1) and in its vicinity (S2—50–200 m, S3—300–400 m, S4—1000 m; Fig.1). They were carried out by means of a Philips PW 3710 X-ray diffractometer (Co kα radiation, 45-kV voltage, 30-mA amperage, impulse counting time 1.2 s, 0.02° step size).

Samples S1, S2, S3 and S4 were subjected also to a high temperature thermomagnetic analysis to determine the major magnetic fractions contributed to the measured magnetic signal. The determination was based on the estimation of the Curie point of dominant magnetic minerals present in the measured material. The analysis were carried out in the temperature range from 20 to 700 °C at a heating rate of 8.5 °C/min using a MFK1 multifunction magnetic susceptibility bridge (AGICO, Brno, Czech Republic), equipped with a CS4 heating chamber. The measurements were conducted both in an air and argon atmosphere.

## Results and discussion

### Mineralogical data

Tailings, like the Zn-Pb-Fe primary ores, have a carbonate mineral composition (Table 1). Among ore-bearing minerals, there are simple sulphides: sphalerite ZnS, PbS galena, marcasite and FeS_2_ pyrite (Kucha and Viaene 1993; Górecka 1996; Viets et al. 1996; Cabala et al., 2004; Sracek et al. 2004). The waste is mainly composed of fine-grained fraction < 0.04 mm (Górecka et al. 1994). They are characterised by high, from 15 to 18%, iron sulphide contents (Cabala et al. 2004; Cabała 2005; Bauerek et al. 2009). XRD analyses proved occurrence of phases which varied concerning their solubility: sulphides, sulphates, carbonates and oxides (Table 1).

**Table 1:** Mineral composition of the top layer (S1) from tailing ponds (semi-quantitative XRD data)

Main gangue components	Dolomite CaMg(CO_3_)_2_, ankerite CaFeMg(CO_3_)_2_, calcite CaCO_3_,	Weak-insoluble
	Illite, montmorillonite, kaolinite, quartz SiO_2_	Insoluble
Sulphides	Marcasite FeS_2_, pyrite FeS_2_, sphalerite ZnS, greenockite CdS,	Soluble
	Galena PbS,	Weak-insoluble
Metalliferous carbonates and silicates	Smithsonite ZnCO_3_, cerussite PbCO_3_	Weak-insoluble
	Hemimorfite Zn_4_Si_2_O_7_(OH)_2_ H_2_O	Insoluble
Sulphates and oxides	Melanterite FeSO_4_ · 7H_2_O, rozenite FeSO_4_ ·4H_2_O, epsomite MgSO_4_ ·7H_2_O, hexahydrite (Mg,Zn,Fe)SO_4_ ·6H_2_O, boyleite (Zn,Mg)SO_4_ ·4H_2_O, bianchite (Zn,Fe^2+^)SO_4_ ·6H_2_O, copiapite Fe_5_(SO_4_)_6_(OH)_2_ ·20H_2_	Readily soluble
	Gypsum CaSO_4_ ·2H_2_O, bassanite CaSO_4_ ·0.5H_2_O, anglesite PbSO_4_, jarosite KFe_3_[(OH)_6_/(SO_4_)_2_],	Weak-insoluble
	Goethite FeO(OH), Mn oxides, baryte BaSO_4_	Insoluble

**Fig. 3:**
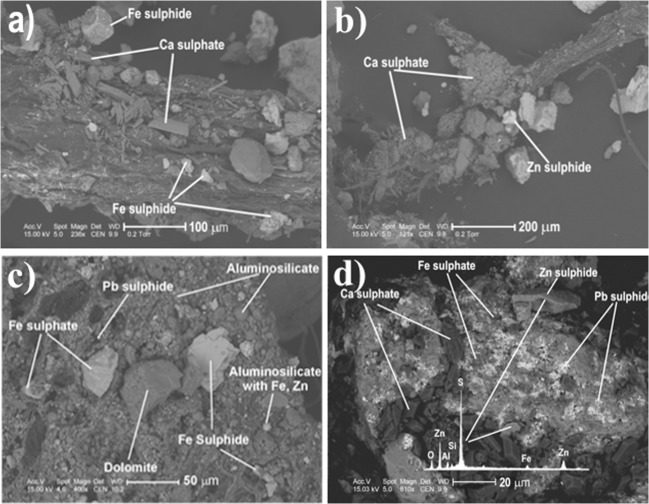
BSE images. **a** Ca sulphates on root epidermis (S2 area). **b** Incrustation of fine-crystalline gypsum on root (S1 area). **c** Fe sulphates in association with FeS i PbS (S3 area) depth 0.2 m. **d** Ca sulphates on Fe sulphates and Pb i Zn sulphides

Among the wastes which build the slopes of the pile, calcium sulphates (gypsum, rarely basanite) are in abundance. Automorphic crystals which sizes vary from a few to 100 μm were identified on the plant roots epidermis (Fig. 3a). Gypsum has built incrustations on roots (Fig. 3b) and aggregates of aluminosilicates and Fe oxides; it occurs in fine-crystalline grains or submicroscopic accumulations in association with Fe and Zn sulphides (Fig. 3d).

Fe sulphates in the form of isolated grains or submicroscopic crystalline accumulations on aluminosilicate and dolomite aggregates were identified in sediments taken from the depth of 0.2 m (Fig. 3c), as well as 0.6 and 0.8 m.

**Fig. 4:**
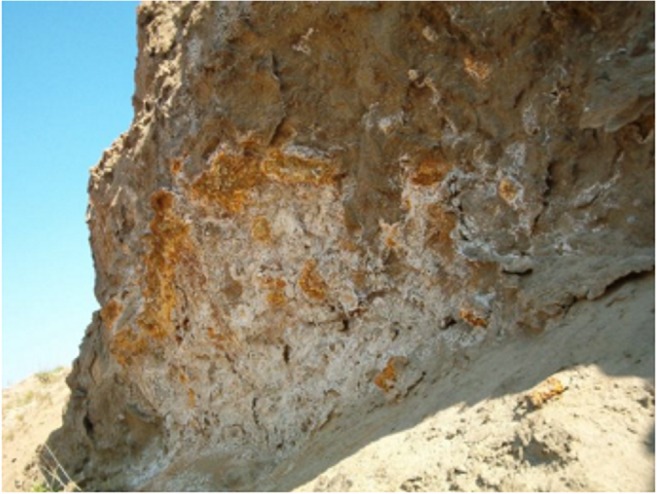
Sulphate minerals on the slope of the tailing pile

Sulphate minerals, represented by gypsum, epsomite, copiapite and bianchite, crystallise in large quantity at the places of water discharge on the pile slopes (Fig. 4). The share of the mineral fraction < 0.04 mm, identified as the material from the pile, is over 5% in the soils around the dumping ground. XRD analyses have shown that on the surface of the pile and in topsoil adjacent to the pile, there is a marked enrichment in Zn-Pb-Fe sulphides, Fe oxides and Zn-Pb carbonates (Table 2). It is important that in sample S1 taken from the initial soil developed on the tailing, the sulphides make up 35% of the mineral composition, with the main sulphides being iron sulphides (marcasite, pyrite). Iron oxides in the waste material constitute only 1%. In soil samples taken outside the pile, the amount of sulphides decreases with the distance. At the same time, the amount of iron oxides increases, reaching 15% in the sample S3 (Table 2). The fine particles containing iron sulphides have been blown out by the wind from the pile and deposited in the soil. Here in oxidising conditions, the sulphides are transformed to iron oxides, which exhibit ferromagnetic properties. This causes the increase of magnetic susceptibility in topsoil in the vicinity of the tailing pile.

**Table 2:** The content of mineral components in the soil from the research area (fraction ϕ < 0.04 mm, values ​​in % determined on the basis of semi-quantitative XRD analyses)

Minerals	S1	S2	S3	S4
Quartz	6	36	25	52
Dolomite & Fe dolomite	33	25	28	8
Kalcite	10	3		5
Markasite	15	10	6	
Piryte & sphalerite	18	9	8	6
Galenite	2		6	1
Cerusite	8			
Smithsonite	5			
Fe oxide & Fe hydrooxide	1	2	15	5
Ca, Zn, Fe, Mg sulphates	2			1
K & Na aluminosilicates		8	3	12
Mullite		5	8	
Kaolinite, illite & mica		2	1	6

S1, S2, S3 and S4 areas of sampling in Fig. 1)

**Fig. 5:**
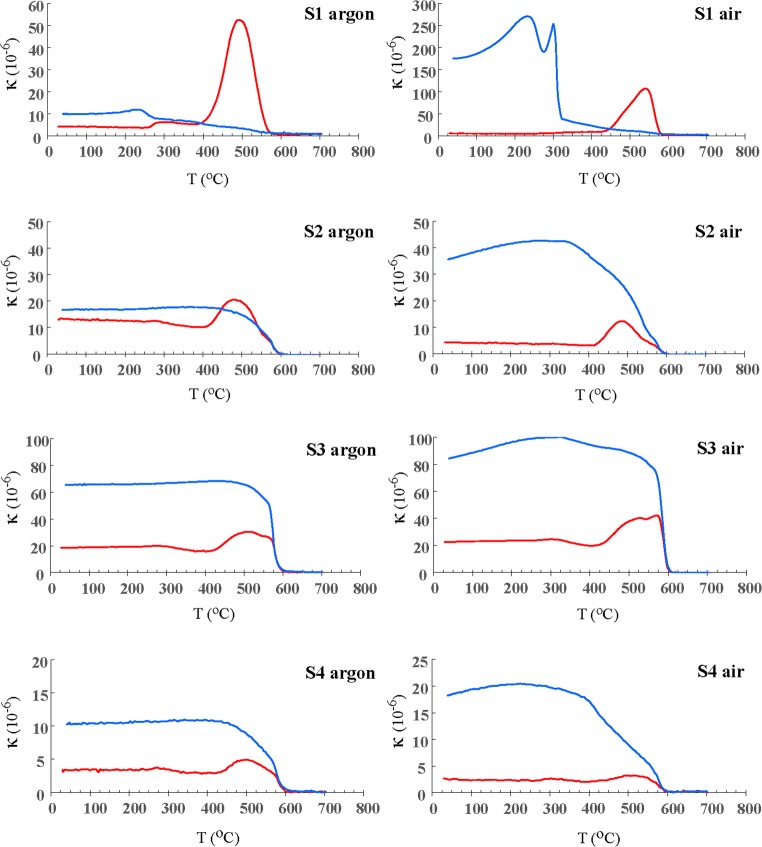
Thermomagnetic curves of samples collected from the waste pile (S1) and from topsoil (S2, S3 and S4), showing changes in magnetic susceptibility with temperature. Measurement conducted in argon and ambient air. Red line, heating curve; blue line, cooling curve

This interpretation is confirmed by thermomagnetic analysis. In the case of sample S1 coming from the pile, the analysis performed in an inert environment (argon) has shown the noticeable increase on the heating curve at approx. 250 °C resulting from the decomposition of sulphides, then a strong peak at about 400 °C where the formation of a ferrimagnetic phase begins (probably a magnetite - Curie point c.a. 580 °C). This phase is unstable and after demagnetization at the Curie temperature retains the characteristics of paramagnetic minerals. During cooling, the sample remains poorly magnetic. At room temperature, the sample has paramagnetic properties and the cooling curve is slightly above the initial κ value of the heating curve (Fig. 5).

The same sample analysed in an oxidising atmosphere behaves differently. The κ value after cooling the sample to room temperature is more than 30 times higher than the initial value, and the sample acquires ferrimagnetic features. The strongly magnetic phase is formed during cooling at a temperature of about 320 °C. This is probably the effect of oxidation of a large amount of sulphides contained in the initial sample, where stable iron oxides form. The same process, although much slower, also occurs in the natural environment in the topsoil, where a fine fraction of sulphides entering the soil due to aeolian transport is subject to oxidation processes. As a result, paramagnetic iron sulphides transform into oxide forms with ferrimagnetic properties causing an increase of topsoil magnetic susceptibility in the vicinity of the pile.

Thermomagnetic analyses of soil samples containing lower content of sulphide minerals did not show such a clear effect of sulphide decomposition. In samples S2 and S4, the cooling curve is slightly above the heating curve, and the low initial and final values of the sample indicate relatively small mineralogical changes occurring in the sample during heating. The small amount of oxides present in the sample is probably represented by magnetite. In the case of heating the samples in air, the cooling curve is slightly above the heating curve, but the increase of the κ value after the heating process is relatively small in relation to the initial value.

A similar pattern is observed in sample S3, showing a higher value of magnetic susceptibility (Table 3). Here, the cooling curve is also above the heating curve and shows a κ value higher in relation to the initial value at room temperature, but the increase in susceptibility in the oxidising environment is not so drastic here (just over 4 times) as compared with sample S1.

**Table 3:** Average magnetic susceptibility (χ) and percentage frequency-dependent susceptibility (χ_FD_ %) of the tailings and soil samples

Sample	*n*	κ (× 10^−5^ SI units)	χ (× 10^−8^ m^3^/kg)	χ_FD_ (%)
Fresh waste	10	14.0	10.0	1.2
S1	10	10.8	8.5	3.1
S2	10	33.7	25.3	4.0
S3	10	91.5	68.5	2.2
S4	10	14.2	10.8	1.2

### Results of geophysical investigations

**Fig. 6:**
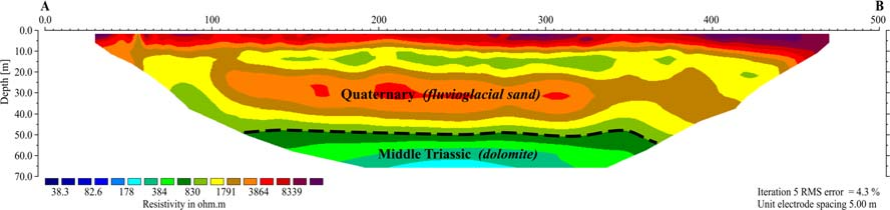
Deep ERT cross section obtained for the A-B profile

The longest line of ERT measurement—A-B profile—enabled to carry out geoelectric recognition to the depth of about 70 m. At the depth of about 50 m, in accordance with the geological data from drilling (Central Geological Data Base 2018), the roof of the Middle Triassic dolomite was interpreted. Its resistivity did not exceed 800 Ωm, (Fig. 6). Above it, there was a complex of high-resistivity Quaternary fluvioglacial sands. In the depth interval of 10–20 m below ground level (bgl), occurrence of sands (probably fine grained) with the resistivity below 2 kΩm was determined. The subsurface layer built of dry sands, whose thickness was up to 10 m, had the highest resistivity up to 8.5 kΩm.

The survey was further continued on the lines A-B′ and C-D, which were 400 m long each. The measurements were performed twice, i.e. during the long dry period (June–July) when the natural humidity of sand amounted several % (Cabała et al. 2007) and in the period of increased humidity of sediments (March).

**Fig. 7:**
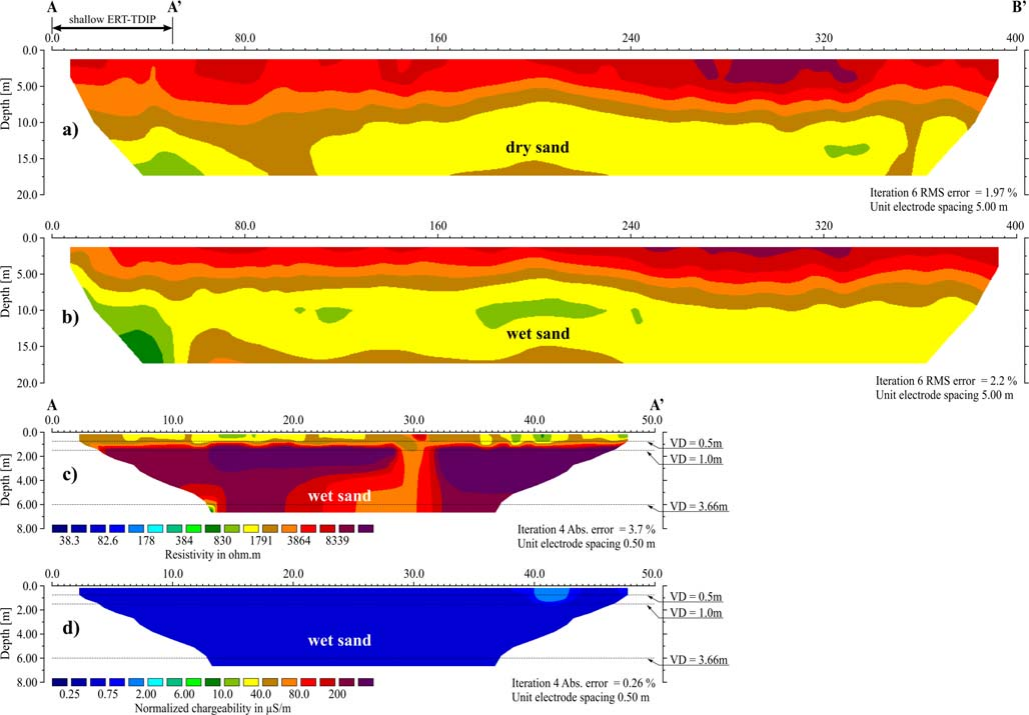
Geoelectrical cross sections for profile A-B. **a** ERT section for the A-B′ interval, dry conditions. **b** ERT section for A-B′ interval, wet conditions. **c** Shallow ERT section for A-A′ interval, wet conditions. **d** Shallow IP section for the A-A′, wet conditions

No significant differences in resistivity were observed in both measurement periods in the cross section A-B′, surveyed in the interval of 0–20 m bgl. The registered resistivities were high, in the range of 0.8–8 kΩm (Fig.7 a and b).

**Fig. 8:**
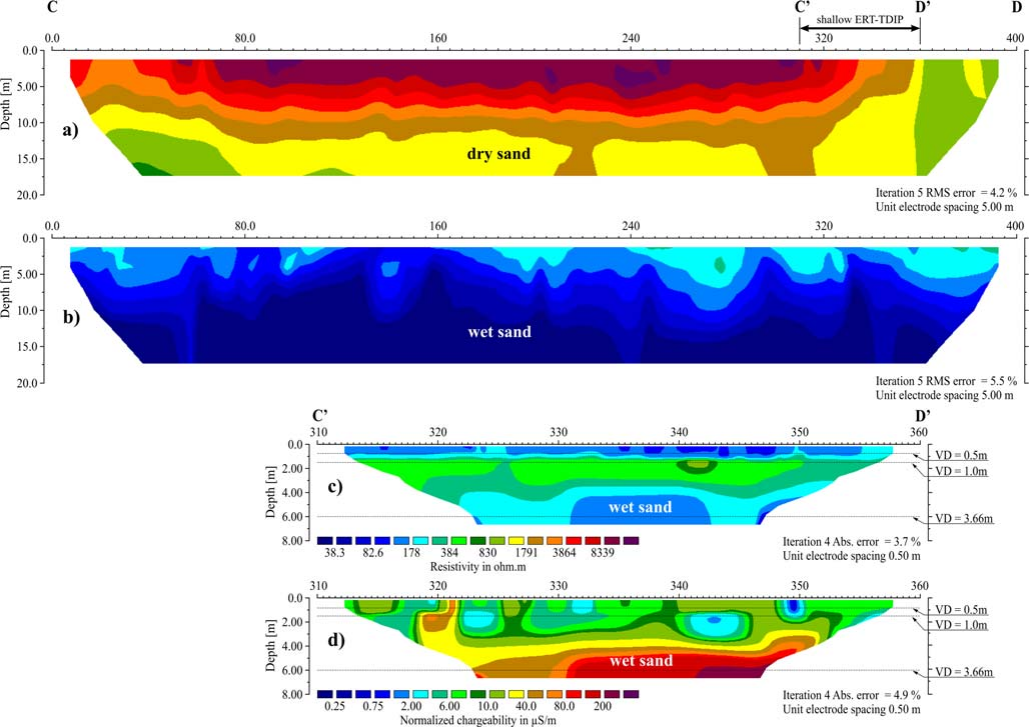
Geoelectrical cross sections for profile C-D. **a** ERT section for the AC-D interval, dry conditions. **b** ERT section for C-D interval, wet conditions. **c** Shallow ERT section for C′-D′ interval, wet conditions. **d** Shallow IP section for the C′-D′, wet conditions

Resistivity survey carried out along the C-D profile showed, in the corresponding depth interval, very strong variation in resistivity depending on the measurement cycle. In dry conditions, the resistivities were identical to those obtained in the A-B′ profile (Fig. 8a), while in high humidity conditions, the measured resistivities were significantly lower and did not exceed 200 Ωm (Fig. 8b).

Due to a relatively large initial distance between electrodes (*a* = 5 m), the thin layers closest to the surface might not correspond to the obtained ERT cross sections. Therefore, in order to trace the variability of the electrical conductivity in the most shallow layer, ERT measurements were supplemented by a shallow-depth electromagnetic survey.

**Fig. 9:**
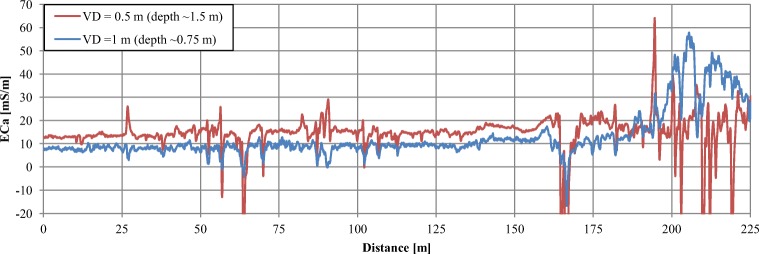
Apparent conductivity EC_a_ along the P-R profile situated on the eastern slope of the tailing pile

In order to determine the conductivity of the tailings, measurements along the 225 m reference profile P-R, on one of the shelves of the eastern slope of the pile, were carried out in the dry period. For both EM-38 depth ranges, up to approximately 180 m of the profile, apparent conductivity values EC_a_ were stable; around 12–18 mS/m to the depth of 0.75 m and about 8–11 mS/m to the depth of approximately 1.5 m (Fig. 9). The material building the slopes of the pile is homogeneous, hence the observed variability may result only from changes in humidity. The average conductivity of dry waste was about 10 mS/m (~ 100 Ωm). In conditions of high humidity, the conductivity of the waste significantly increased, reaching even 250 mS/m (~ 4 Ωm) (Pierwoła, 2015). The zone of strong disturbances, visible at the end of the profile at both depths, correlated with the traces of mass movements on the slope.

**Fig. 10:**
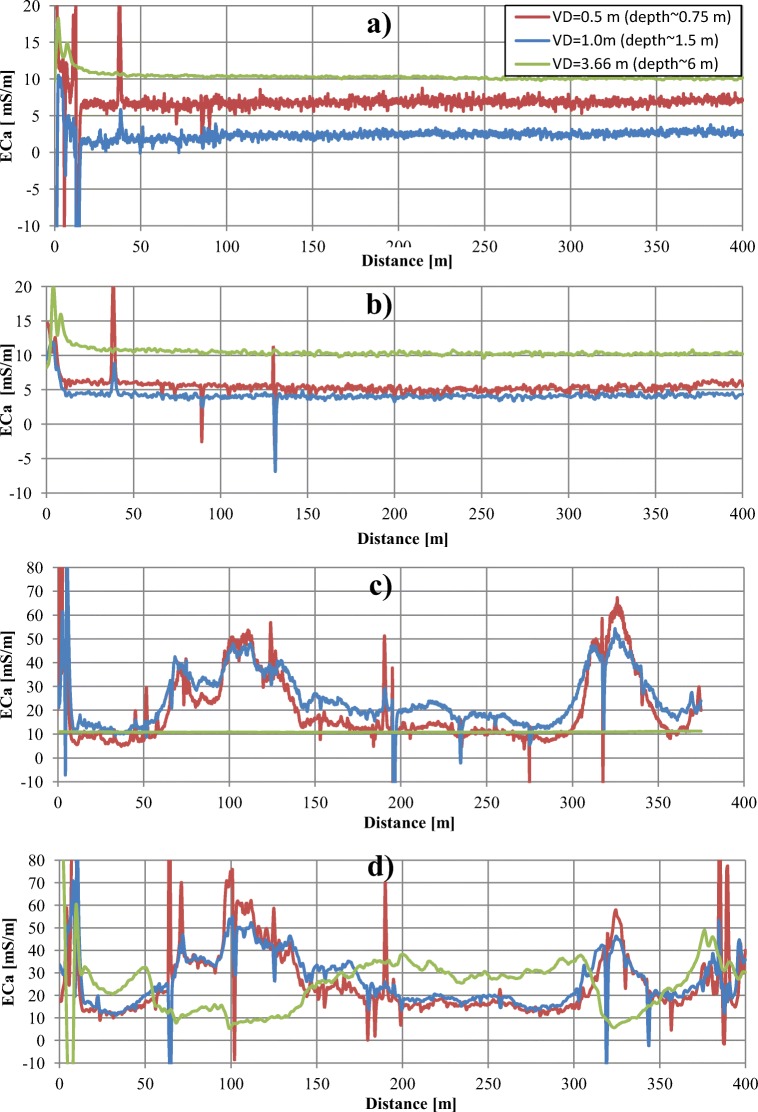
Apparent conductivity EC_a_ for search lines. a A-B′, dry conditions. b A-B′, wet conditions. c C-D, dry conditions. d C-D, wet conditions

Conductometric measurements enabling the survey of distribution of the EC_a_ of sands in three shallow-depth intervals (0–0.75 m, 0–1.5 m and 0–6 m) were performed, like the resistivity ones, in two cycles (Fig. 10).

The EC_a_ measured along the A-B′ profile in dry conditions was about 5 mS/m lower than the ones obtained in the P-R profile, and its values were approximately 6–7 mS/m in the depth interval of 0–0.75 m, 2–3 mS/m in the interval of 0–1.5 m and 10–11 mS/m in the interval of 0–6 m (Fig. 10a). In all depth intervals, a significant increase of conductivity when compared with the background was observed within the first 20 m of the distance, i.e. in the part closest to the pile. The highest amplitude of the disturbance was observed in the curve of the shallowest profiling, hence it can be assumed that the cause of its occurrence was located just below the ground surface. Measurements repeated during the humid period (Fig. 10b) did not show any significant changes in conductivity.

Due to technical reasons, it was necessary to shorten the C-D line by 25 m in relation to the length of the ERT line during the dry period. In that profile, EC_a_ in the two shallow-depth intervals (Fig. 10c) was significantly higher (5–55 mS/m) in dry conditions comparing with the values obtained in the A-B′ profile, and they were close to the values from the P-R lines. The deepest profiling showed EC_a_ values similar to those obtained on the A-B′ line. In two shallow-depth intervals of the C-D profile, the amplitude of conductivity changes reached 60 mS/m; EC_a_ maxima were observed between 65 and 145 m and 305 and 355 m. The zones of conductivity maxima were recorded in the places of visible traces of landslides on the slopes of the pile. In the parts of the profile located outside the abovementioned anomalous zones, the highest EC_a_ was recorded within the depth interval of 0–1.5 m.

Contrary to the A-B′ line, a rise in EC_a_ was observed along the C-D profile (Fig. 10d) in the wet period; for two shallow-depth intervals, it varied from a few to approximately 20 mS/m, without any changes of the primary shape of the curves; for the deepest interval (0–6 m), it was 20–30 mS/m, with a significant change of the shape of the obtained curve. Maxima on the curves obtained from the shallow-depth profiling correlated with the minima on the curve from the deeper profiling.

During the further stage of the investigation, shallow-depth measurements of the induced polarisation were performed in conditions of increased humidity, in the selected zones of the maximum EC_a_ on both conductometric lines. The line A-A′ was determined between 0 and 50 m of A-B′ and the C′-D′ line between 310 and 360 m of C-D. The depth range was about 6 m, which corresponded to the deepest conductometric survey. The obtained sections, despite the measurement being performed in stable conditions, differed from each other in the level of observed resistivity (Figs. 7c and 8c) and normalised chargeability (measure of IP effect) (Figs. 7d and 8d). In the section A-A′ the resistivity did not drop below 500 Ωm, and the normalised chargeability (MN) was very low (< 0.5–0.75 μS/m), while along the C′-D′ line, the resistivity did not exceed 500 Ωm and the MN was 2–40 μS/m to the depth of 3 m, and deeper it increased to approximately 200 μS/m.

Surface measurement of low-field magnetic susceptibility (κ) of the soil (depth 0.1 m) carried out in the dry period was the last stage of the survey. The magnetic susceptibility was measured along the profile A-B on the distance of 400 and between 0 and 200 m of the profile C-D. The last 200 m of the profile C-D was not suitable for the surface magnetic measurement due to the local puddles and sawmill waste lying on the surface. Along the A-B profile, measurements were taken in 3 transects at a distance of 10 m from each other. In the case of C-D profile, measurements were carried out only in 2 transects due to the specific field conditions.

**Fig. 11:**
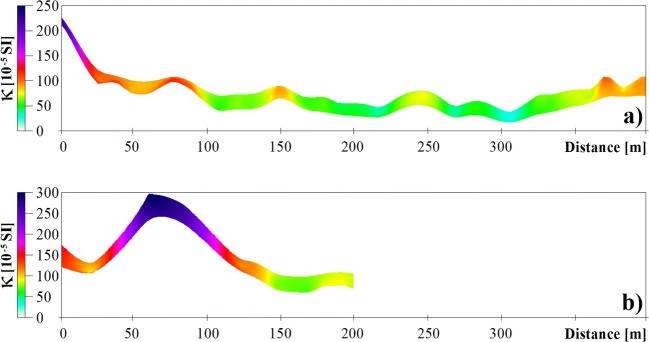
Spatial distribution of mean values of the volume magnetic susceptibility (κ) for A-B (**a**) and C-D (**b**) search lines

In the A-B profile (Fig. 11a) located in the open ground, east of the pile, the highest magnetic susceptibility values ​exceeding 200 × 10^−5^ SI magnetic units occurred in the immediate vicinity of the pile. The value of κ was about 100 × 10^−5^ SI units over a distance of 25–75 m, while further up to 300 m from the pile, it fluctuated between 30 and 90 × 10^−5^ SI units. Further than 300 m, the value of κ began to grow again, reaching 100 × 10^−5^ SI units at a distance of 375–400 m from the pile. The increase has been associated with the morphology of the area (increase of the elevation).

In the western part of the C-D transect (0–40 m), the magnetic susceptibility values remained in the range of 100–150 × 10^−5^ SI units. In the further part of the profile (along the 40–120-m stretch), there was a rapid increase in the value of κ up to 300 × 10^−5^ SI units, and then decrease of the values. From 140 m, the κ values remained in the range of 60–100 × 10^−5^ SI units. A double increase of the κ value (between the 40 and 120 m) corresponded to the fragment of the C-D profile, where a significant increase of relative conductivity on the curves of the shallow-depth electromagnetic profiling was observed (there are visible effects of landsliding processes on the slope of the pile).

## Discussion

The grounds where tailings of metal ore processing have been dumped impact the environment as a result of various processes: chemical, biological, aeolian, water flow, etc. The purpose of conducting the comprehensive geophysical survey was to test possibilities to detect the most important ones: deep infiltration of mineralised water from the tailing pond through the dumped material, rapid migration of material as a result of surface run-off caused by heavy rains and aeolian transport of the smallest fractions deposited on the pile.

The results of the conducted electrical resistivity and electromagnetic surveys indicate strong response caused by changes of humidity of fluvioglacial sands adjacent to the tailing pile in the north and the lack of such response in the area located east of the pile. Natural lithological variation of the surveyed material as a possible reason of the observed changes should be excluded.

To interpret geophysical surveys properly, it is advisable to identify the area in terms of its mineralogy and geochemistry, which has been confirmed by the results of the earlier works in the areas contaminated by historical wastes from processing of Zn-Pb ore of the MVT type (Cabala et al. 2008).

Carbonate minerals (dolomite and calcite) are the dominating component of the tailings constituting about 73% of the waste, while clay minerals constitute approximately 10%, Zn and Pb carbonates < 1%; the share of Fe sulphides (pyrite and marcasite) (approx. 15–18%) is also significant (Cabala et al. 2004, 2008). Unstable sulphides (FeS_2_, ZnS, PbS) and dolomite are the main source of metal ions and sulphates in the migrating waters from the tailings.

Sulphate ions are mobilised, and the Fe^2+^, Zn^2+^ and Mg^2+^ ions are activated in the oxidation reactions of FeS_2_ (1) and ZnS (2). The process is less intensive in the case of Pb^2+^.

1 $$ {\mathrm{Fe}\mathrm{S}}_2+3.{5\mathrm{O}}_2+{\mathrm{H}}_2\mathrm{O}=>{\mathrm{Fe}}^{2+}+{{2\mathrm{SO}}_4}^{2-}+{2\mathrm{H}}^{+} $$

2 $$ \mathrm{ZnS}+{2\mathrm{Fe}}^{3+}+1.{5\mathrm{O}}_2+{\mathrm{H}}_2\mathrm{O}=>{\mathrm{ZnSO}}_4+{2\mathrm{Fe}}^{2+}+{2\mathrm{H}}^{+} $$

The Ca^2+^, Mg^2+^, Fe^2+^ and Zn^2+^ ions which are present in the waters enable stabilisation of SO_4_^2−^ in hydrous sulphates of Ca (gypsum), Mg (epsomite), Fe (melanterite, copiapite, rozenite) and Zn (bianchite). Fe^2+^ ions are stabilised in Fe oxides and hydroxides (limonites), which form ocher in the shallow layers of the soil. In the carbonate environment of the tailing pile with water deficit, unstable Zn sulphates quickly turn into carbonates (3), and sulphate ions are bound in gypsum.

3 $$ {\mathrm{ZnSO}}_4+{\mathrm{CaCO}}_3=>{\mathrm{ZnCO}}_3+{\mathrm{CaSO}}_4 $$

The processes of leaching and dissolving of sulphides deposited in the tailing pile occur relatively quickly; the process of dissolving hydrated sulphate phases (melanterite, bianchite, goslarite or gypsum) is even faster. The sulphates, in the form of dispersed crystalline grains were found in large amount in the soils around the pile. During the periods of increased humidity, some of the SO_4_^2−^ and heavy metal ions infiltrate into the permeable sandy sediments together with the surface run-off waters. The ions Zn^2+^, Pb^2+^, Cd^2+^, Tl^+^ and As^3+^ and also SO_4_^2−^ have been released into solutions from metal-bearing minerals, and soils have been contaminated with them (Jambor et al. 2000). Organic matter of the soil causes additional release of OH^−^ ions, humic, fulvic acids and others.

Despite the fact that the carbonate type of the wastes and bedrock limits the migration of acid solutions, the presence of Fe, Zn and Pb sulphides higher than 15% makes the development of AWD still possible (Cabała 2005).

High buffering properties of post-flotation wastes considerably reduce leaching of SO_4_^2−^, Zn^2+^, Ca^2+^, Mg^2+^ and Fe^2+^ ions (Postawa and Motyka 2018), but their quantity transferred from the dump remains high. Motyka et al. (2005) showed that about 5.0 m^3^ of leachate containing, on average, 1400 mg/dm^3^ of sulphates infiltrates per minute from the tailing ponds to the underlying sediments. The average sulphate content in the water drained by the Pomorzany mine is up to 300 mg/dm^3^ (Różkowski et al. 1997). About 1.42 mg/dm^3^ of zinc; up to 0.32 mg/dm^3^ of lead; and significant amounts of cadmium, copper, arsenic and nickel are transferred per minute from the pile to the ground (Motyka et al. 2005).

The favourable conditions for the development of acid waste drainage occur in the presence of increased humidity in the investigated area. Significant changes in resistivity/conductivity depending on atmospheric conditions should be interpreted as the result of the occurrence of easily soluble sulphates beyond the tailing pile. The most important reason for the transfer of sulphates is probably inclination of the slopes of the pile, which, with an average precipitation of about 600 mm/year, enable rapid gravitational outflow of rainwater outside the pile boundary.

Laboratory tests of tailings (Cabała et al. 2007) show that the relation of conductivity and humidity is an exponential function, which indicates high levels of easily soluble minerals. Surface run-off water is characterised by mineralisation 300–600 mg/dm^3^, which is mainly caused by sulphates (about 400 mg/dm^3^) (Bauerek et al. 2009). It results in high electrical conductivity of the running off water, amounting to an average of 525.4 μS/cm (≅ 19 Ωm). The water penetrating into the sandy sediments carries acidic products of the drainage, which in the vadoze zone of fluvioglacial sands cannot be buffered in the form of poorly soluble carbonate minerals. As a consequence, hydrous Fe, Mg, Zn and Ca sulphates crystallise. In the periods of increased humidity, sulphates are dissolved in pore spaces, forming electrolytes, which has been reflected in the results of ERT and EM surveys.

The routes of hydraulic transport on the surface of the pile slopes are clearly marked by occurrence of white and yellowish accumulations of sulphate minerals (Fig. 4). The zones of peaks of apparent conductivity (C-D) in shallow conductometric profiles correlate with occurrence of the material sliding down the slopes of the pile. Hence the reduced resistivity, comparing with the A-A′ cross section and increased chargeability as well as increased magnetic susceptibility κ (Fig. 11b) associated with secondary iron oxide minerals (Cabała 2005; Table 2) can be interpreted as a result of penetration of fine-grained fractions of the waste material into the layer of fluvioglacial sands at the foot of the pile.

One of the ecological problems of newly formed or old, but poorly turfed tailing piles, is the dusting process. Taking into account the fact that tailings are composed mainly of fine grains < 0.04 mm, the phenomenon can be very intense. Blowing away of fine waste fractions containing various sulphides has led to the spread of the latter in soils in the areas adjacent to the pile. The processes of chemical transformation of Fe sulphides have led to formation of new ferri- or antiferromagnetic phases such as maghemite and haematite. A fine fraction of secondary iron oxides has been removed together with other mineral particles from the unturfed fragments of the pile, and it has been transported by air, resulting in increased magnetic susceptibility of the soils around the pile. Considering low magnetic susceptibility of material (referred to local background) and soils developed on it (with dia- or paramagnetic properties; Łukasik et al. 2016), the increase of magnetic susceptibility in topsoil is due to the deposition of dust containing secondary iron oxides from dusting source (Szuszkiewicz et al. 2016; Jordanova 2016). Hence, the magnetic susceptibility in topsoil is decreasing with the distance from this source. Morphology of the area influences the measured value of κ of the surface layer of soil, causing increased value of κ on local elevations or slopes inclined towards the source of pollution (Fig. 11a). A similar effect was also observed in the area of ​the post-industrial waste dumping ground of zinc and lead smelter in Piekary (Rachwał et al. 2017).

Aeolian transport may also be the cause of increased apparent conductivity of the most shallow (VD = 0.5 m) soil layer. Tests of the soil samples taken at the sampling points S1, S2 and S3 (Fig. 1) arranged in accordance with the wind direction dominating in the test area have shown more or less constant content of minerals transported by wind from the pile up to the distance of about 500 m from it. The share of metalliferous minerals decreases with the increase in depth and distance from the pile; however, their occurrence in the topsoil indicates an important role of wind in the redeposition of grains and mineral aggregates in the zone up to approximately 1.5 km from the tailing pile (S4).

## Conclusions

Integrated geophysical and geochemical methods applied in this study enabled identification of different threats related to underground water infiltration, acid waste drainage and dusting processes. Correct identification of zones contaminated with unstable metalliferous minerals should be carried out using the complex of methods: FDEM, ERT, TDIP and soil magnetometry. The results obtained on the basis of the combined geophysical, mineralogical and geochemical methods have shown the range of the impact of ecological changes around the tailing pile. The changes in the chemical properties of the soils around the tailing pile depend not only on the directions and dynamics of the water flow from the pile but also on the aeolian transport responsible for the redeposition of substantial quantities of fine-grained fractions of waste enriched with unstable sulphides, and what more, it was able to identify zones of pollution spreading around the tailing pile.

The geochemical and mineralogical studies revealed the presence of easily soluble, secondary, sulphate mineral phases in the deposited wastes and in their vicinity, which indicates that their crystallisation had been derived from solutions originated in AWD processes. The crystalline sulphate phases Ca, Fe, Mg and Zn occurring in the soils and the sands around the tailing pile are mineralogical markers of the migration routes of the pollutants. Occurrence of unstable, metal-bearing phases is the evidence of the migration of ephemeral electrolyte-type solutions that have a significant impact on the geoelectric and magnetic properties of the rock medium.

The following procedures of geophysical methods have been proposed:

- Induction profiling as a quick and inexpensive method in the initial stage of the study to design the scheme of the further measurements and location of geophysical profiles.
- Soil magnetometry and shallow-depth induction profiling for identifying a zone contaminated with minerals redeposited by aeolian processes and assessing the dusting range of the pile.
- Electrical resistivity methods to assess the range of the zones changed by AWD products and directions of migration pollutants. As this method is strongly dependent on humidity of the sediments, it would be recommended to run the test in at least two cycles (humid and dry periods).
